# Lignin–carbohydrate complexes: properties, applications, analyses, and methods of extraction: a review

**DOI:** 10.1186/s13068-018-1262-1

**Published:** 2018-09-29

**Authors:** Dmitry Tarasov, Mathew Leitch, Pedram Fatehi

**Affiliations:** 1 Chemical Engineering Department, Lakehead University, 955 Oliver Road, Thunder Bay, ON P7B 5E1 Canada; 2 Natural Resource Management Faculty, Lakehead University, 955 Oliver Road, Thunder Bay, ON P7B 5E1 Canada

**Keywords:** Lignin–carbohydrate complex (LCC), Sustainable chemicals, Milled wood lignin, NMR, Fractionation

## Abstract

The complexity of lignin and hemicellulose segmentation has been known since the middle of the ninetieth century. Studies confirmed that all lignin units in coniferous species and 47–66% of lignin moieties in deciduous species are bound to hemicelluloses or cellulose molecules in lignin–carbohydrate complexes (LCC). Different types and proportions of lignin and polysaccharides present in biomass lead to the formation of LCC with a great variety of compositions and structures. The nature and amount of LCC linkages and lignin substructures affect the efficiency of pulping, hydrolysis, and digestibility of biomass. This review paper discusses the structures, compositions, and properties of LCC present in biomass and in the products obtained via pretreating biomass. Methods for extracting, fractionating, and analyzing LCC of biomass, pulp, and spent pulping liquors are critically reviewed. The main perspectives and challenges associated with these technologies are extensively discussed. LCC could be extracted from biomass following varied methods, among which dimethyl sulfoxide or dioxane (Björkman’s) and acetic acid (LCC-AcOH) processes are the most widely applied. The oxidation and methylation treatments of LCC materials elucidate the locations and frequency of binding sites of hemicelluloses to lignin. The two-dimensional nuclear magnetic resonance analysis allows the identification of the structure and the quantity of lignin–carbohydrate bonds involved in LCC. LCC application seems promising in medicine due to its high anti-HIV, anti-herpes, and anti-microbial activity. In addition, LCC was successfully employed as a precursor for the preparation of spherical biocarriers.

## Background

Biomass shows great potential for fuel and non-fuel applications. It is a mixture of cellulose, hemicellulose, lignin, and extractives [1], which are considered as the most common natural polymers on earth [2].

Lignin, hemicellulose, and cellulose form unique and complex structures in wood. Softwood species include 33–42% cellulose, 22–40% hemicellulose, 27–32% lignin, and 2–3.5% extractives [3, 4]. Hardwood species contain 38–51% cellulose, 17–38% hemicellulose, 21–31% lignin, and 3% extractives [4, 5]. The amount of lignin, hemicelluloses, cellulose, and extractives in herbaceous plants are 0–40%, 20–50%, 25–95%, and 4–9%, respectively [5–7].

These polymers are widely applied for manufacturing different products. For example, hemicelluloses are used for ethanol or xylitol production [8, 9]. Lignin is used for producing carbon fibers and dispersants [10, 11]. Cellulose is used in pharmaceuticals and the papermaking industry [12–14].

To produce value-added products, biomass’ components should be separated. Hydrolysis, pulping, and bioconversion processes are considered the dominant fractionation processes of biomass. Hydrolysis aims at separating hemicelluloses from other components of biomass. Pulping processes are common methods to obtain cellulosic materials used for producing various paper grades. Bioconversion procedures can also be applied for separating cellulosic sugars from biomass. These methods are based on the liberation of the bonds of lignin and holocellulose.

Despite its effectiveness, biomass fractionation is characterized by some challenges, one of which is the difficulty in separating lignin from carbohydrates [15, 16]. Lignin and carbohydrate moieties are chemically bound in native biomass forming a lignin–carbohydrate complex, LCC [17, 18]. LCC linkage plays a crucial role in wood structure, since all lignin moieties in softwoods [19] and 47–66% of lignin fragments in hardwoods [20] are bound to carbohydrates, mainly to hemicellulose [21]. Numerous studies report the presence of LCC in native biomass materials in coniferous, deciduous, and non-wood plants [19, 22–25]. Due to its strong bonding, the presence of LCC affects the overall extraction of lignin and carbohydrates [16, 24, 26]. For example, a low yield of kraft pulping process is related to the challenge in breaking lignin–carbohydrate linkages in hardwood species [27], which can be attributed to the alkaline stability of LCC bonds. In addition, the formation of lignin–hemicellulose linkages in kraft pulp has been suggested [28, 29]. Several studies confirm the existence of lignin–hemicellulose linkages in softwood [30] and in hardwood kraft pulps [20, 31]. Chen et al. [32] report that autohydrolysis of hardwood results in extracting xylan in the initial stage of autohydrolysis, whereas the isolated xylan units are found to be associated with lignin in the later stage of autohydrolysis. Another study reports that the efficiency of enzymatic hydrolysis of poplar is significantly affected by LCC linkages [33]. Carbohydrates are covalently anchored and shielded by lignin in plant cell walls, which reduces the area of cellulose accessible for enzymatic attacks [34, 35]. The cleavage of LCC bonds is reported to improve the enzyme accessibility to biomass [36, 37]. In addition, the existence of covalent cross-linkages in forage grasses significantly affects the ability of ruminants to digest, due to the limited access of rumen fermentation microorganisms to carbohydrates in the fodders [36]. Therefore, a better understanding of LCC structure may help to determine appropriate processes to break lignin–carbohydrate bonds, and thus to extract lignocelluloses from biomass effectively and selectively [18].

The molecular weight (MW) of lignin is reported to be an essential parameter for its application as a flocculant and dispersant [38]. The low content of methoxyl groups reduces the heat capacity (*C*_p_) of lignin and increases its glass transition temperature (*T*_g_) [39, 40]. The heating values of lignocellulosic material are, to a large extent, dictated by the presence of inorganic compounds [41], which also affect the *T*_g_ value of lignin [42]. Lignin with a low *C*_p_ and a high heating value can be utilized as fuel or a binder for pellet production [42]. The hydrophilicity and structural plasticity of lignin is reported to have a positive correlation with its phenolic groups [39, 43].

The properties of hemicelluloses and cellulose also impact their applications. It is reported that low-molecular-weight (MW) sugars are favorable for biofuel production [44, 45], while hemicelluloses with a high MW can be used in cosmetics and pharmaceutical products [46, 47]. Moreover, high MW polysaccharides can be employed in the food industry. For example, galactoglucomannan obtained from process waters of thermomechanical pulping process demonstrates an MW between 39,000 and 46,000 g/mol [48] and can be applied as a replacement for gear or xanthan gums [47]. However, the relatively low heating values of hemicelluloses and cellulose [49, 50] limit their applications as fuel.

According to our knowledge, there is no report available to discuss the properties of LCC and their impact on its end-use applications. This study intends to (1) introduce LCC and its properties, (2) describe methods followed in literature to produce LCC, (3) describe the methods used to quantify and analyze the compositions, structures, and properties of LCC, and (4) review the proposed LCC applications.

## Lignin–carbohydrate linkages

In 1838, Paymen proposed an “incrustation theory”, which assumes that lignin crusted cellulosic materials. The “incrustation theory” is based on the observation that cellulose in the cell walls has different properties when non-cellulosic materials are isolated from wood [51]. Erdmann [52] explained the complexity of disuniting lignin from carbohydrates by the fact that these polymers were associated with “glycolignose” materials [53]. Recent research has confirmed that lignin and hemicelluloses are covalently bound and form lignin–carbohydrate complexes [1, 54]. There is now more information about the bonds between lignin and cellulosic molecules. Eriksson et al. [55] suggests a fractional bonding between lignin and cellulose units in softwoods. Lam and Iiyma [56] propose lignin–cellulose linkages in rice straw. Jin et al. [54] report that over 50% of lignin units in softwoods and 17% in hardwoods are covalently (molecularly) bound to cellulose moieties in wood. The linkages between lignin and pectin units are also suggested in wood [57, 58].

**Fig. 1:**
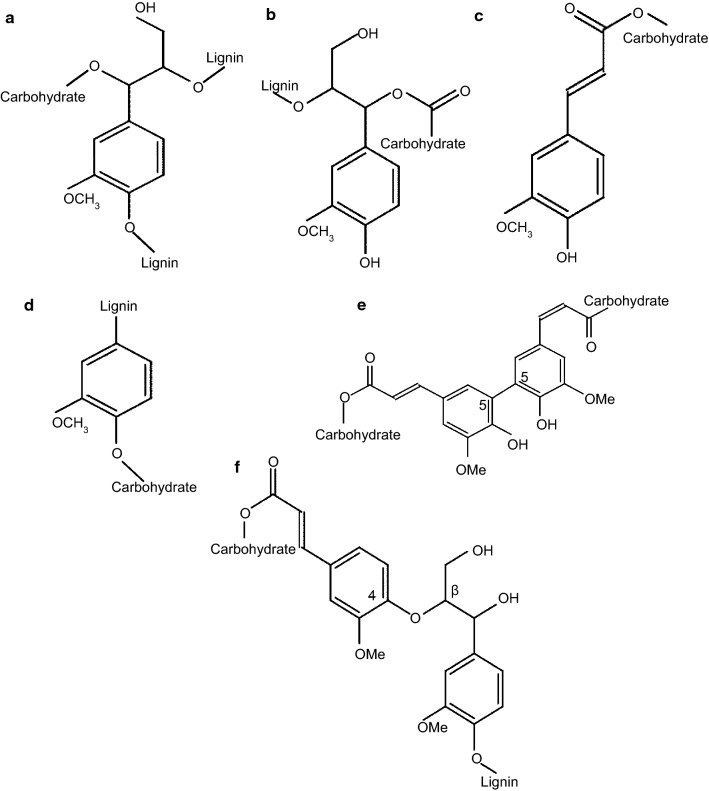
Main types of LCC linkages: **a** benzyl ether; **b** benzyl ester; **c** ferulate ester; **d** phenyl glycosidic; **e** diferulate ester (5′–5′ linkage) **f** diferulate ester (4-*O*-β linkage) (after Ref. [62])

Linkages between lignin and carbohydrates are generated under the conditions of lignin biosynthesis. During nucleophiles supplement to quinone methides, the intermediate connections are developed due to *p*-hydroxycinnamyl alcohol oxidation [59]. There are eight different types of lignin–carbohydrate (L–C) bonds, i.e., benzyl ether, benzyl ester, glycosidic or phenyl glycosidic, hemiacetal or acetal linkages, and ferulate or diferulate esters that are linked to lignin at 4-OH and 4-O positions [37, 55, 60–62]. Figure 1 presents the structures of the main types of LCC bonds. Benzyl ester bonds connect lignin and carbohydrate moieties through uronic acid of sugars and hydroxyl group of lignin; benzyl ether and phenyl glycosidic link glycosyl or mannosyl residues of carbohydrates and phenolic or hydroxyl groups of lignin [53, 62]. Glycosidic bonds link carbohydrates and side chain hydroxyl groups of lignin [37]. Acetal bond is the linkage generated by the carbonyl groups of phenylpropane structural fragments of lignin and hydroxyl groups of carbohydrates [51]. Ferulate and deferulate esters present the major part of LCC linkages in grasses and other non-wood plants [37]. It has been found that high amounts of ferulate and coumarate acids are bound to carbohydrates in cell walls in different herbaceous plants [62]. Ferulic acid demonstrates the ability to oxidatively couple with lignin, proteins, and other ferulic acids [63]. Due to the presence of carboxylic acid groups at the end of propenyl groups, ferulate acid is able to produce ester linkage with polysaccharides [63]. Consequently, ferulate esters of polysaccharides are linked with lignin via oxidative coupling and form “lignin–ferulate–polysaccharide” (LFP) complexes [24, 64]. Due to the abundance of ferulic acids in herbaceous plants and lack of information about ferulic acid existence in the wood fibers, there are limited studies about ferulate linkages in wood. The bark of softwood materials is reported to possess some ferulate esters [65]. Reiter et al. [66] propose ferulic acid formation in the kraft pulping process due to the cleavage of aryl parts of ethers.

Benzyl ether (BE), ester, and phenyl glycosidic (PhyGlc) are the most typical lignin–carbohydrate linkages [21, 62], showing varying strengths under different conditions. Benzyl ether bonds are reported to be alkaline-stable [53, 59]. However, benzyl ether linkages with phenolic hydroxyl groups are proposed to be alkali-liable [67].

Benzyl ester linkages are easily cleaved in alkaline conditions [53, 59]. Silva et al. [27] report that hardwoods with a high content of PhyGlc linkages show the lowest kraft pulping yield performance, which could be related to the alkaline-stable nature of the PhyGlc bonds [68, 69]. In another study, it is noted that the hydrolysis of PhyGlc bond leads to only 4% cleavage in neutral aqueous conditions, whereas 96% of the bond can be cleaved via acid treatment, which is proposed to be generated during hydrothermal treatment of hardwood species [70]. Consequently, a high acid liability and alkali resistance of PhyGlc linkages are suggested. Benzyl ether and ester bonds are also not stable under acid hydrolysis conditions [67, 71].

Ferulate esters are highly alkali-liable and can be cleaved by alkaline hydrolysis at room temperature [53]. However, due to the affinity of ferulate acid to generate ester bonds with hemicelluloses and ether linkages with lignin, the alkali treatment of herbaceous plants results in the extraction of lignin and ferulic acid molecules [53, 72].

## Quantity of lignin–carbohydrate bonds in wood and non-wood species

It can be postulated that the benzyl ether bonds are dominant in softwood LCC [17, 21, 73]. The phenyl glycosidic linkages are prevalent in hardwood LCC, while the amount of ester linkages greatly varies in different species [21, 74]. Herbaceous plants show significantly higher amounts of LCC linkages than woody materials. The quantification of lignin–carbohydrate linkages in herbaceous plants demonstrates the existence of a vast majority of phenyl glycosidic linkages in LCC [23, 24]. The quantification of lignin–carbohydrate linkages in various LCC is presented in Table 1. Each lignin monomeric unit contains aromatic rings, which is equivalent to mol percentage of aromatic hydrogen or carbon atoms [18, 75–77]. The estimation of lignin’s substructures and LCC bonds is mostly expressed as number per 1 or 100 aromatic units (Ar) [21, 75, 76].

**Table 1:** Quantification of lignin–carbohydrate linkages in LCC

LCC preparation	Amounts of LCC linkages per 100 Ar		
	Benzyl ether	PhyGlc	γ-Ester
Softwood LCC			
Pine LCC-AcOH [21]	4.3	6.8	4.5
Spruce GM–L [17]	3.8	4.4	ND
Spruce Xyl–L [17]	6.1	ND	ND
Hardwood LCC			
Birch LCC-AcOH [21]	0.7	5.4	5.6
Eucalypt LCC-AcOH [74]	0.3–0.9	5.0–7.9	ND
Herbaceous LCC			
A. Donax Björkman LCC [24]	251	42	3
Wheat Björkman LCC [23]	50	2	14
A. Donax LCC-AcOH [24]	4	ND	0.14

## Softwood LCC structure

Table 2 depicts the compositions of LCC fractions extracted from various biomass sources. There are a few hypotheses about the LCC structures and compositions for softwoods. Balakshin et al. [21] stated that the benzyl ether linkages in softwood LCC mainly involve mannose. In another study, Giummarella et al. [73] suggest that xylose is the main carbohydrate with ether bonds, whereas mannose is linked by PhyGlc bonds. Lawoko et al. [19] suggest that lignin involved in LCC has two different structures: one type attached to xylan and the other one connected to glucomannan. It is proposed that xylan is linearly linked to lignin, while GM–L complexes have branched structures [19].

**Table 2:** Compositions of LCC fractions from wood and herbaceous plants

LCC fraction	Relative composition of LCC fraction, %		Relative carbohydrate composition, %				
	Acid-soluble lignin	Total carbohydrates	Ara	Xyl	Man	Gal	Glu
Softwood (*Spruce*) [19]							
GGM–L–Pectin	39	56.9	5.6^a^	4.2^a^	58.9^a^	22.1^a^	9.1^a^
GM–L–Xyl	56	36.8	3.3^a^	8.4^a^	52.2^a^	10.9^a^	25.3^a^
Glu–L	7	89.8	1.1^a^	1.0^a^	2.8^a^	0.22^a^	94.9^a^
GM–L–Xyl	41	51.8	1.3^a^	6.2^a^	64.7^a^	3.5^a^	24.1^a^
Xyl–L–GM	65	32	9.7^a^	58.4^a^	13.4^a^	7.2^a^	11.2^a^
Xyl–L–GM	29	59.1	6.9^a^	59.0^a^	24.0^a^	7.11^a^	2.9^a^
Softwood (*Spruce*) [17]							
Glu–L	19.3	80.7	1.9	2.5	8.6	1.2	85.8
GM–L	29.2	70.8	4.7	10.6	30.9	4.4	49.4
Xyl–L	42.7	57.3	13.0	65.3	3.2	3.0	15.6
Hardwood (*Eucalyptus globules*) [31]							
Glu–Xyl–L	14.2/15.8^a^	85.8/84.2^a,b^	0.6/0.7	18.1/18.9	2.3/2.2	1.3/1.5	77.2/76.3
Xyl–Glu–L	34.7/39.6^a^	60.4/65.3^a,b^	2.0/2.2	42.7/44.4	2.9/3.3	4.1/4.9	44.6/47.1
Hardwood (*Eucalyptus globules*) [20]							
Xyl–Glu–L	29.0	71^c^	1.2	40.8	10.4	8.0	39.6
Glu–Xyl–L	53.2	46.8^c^	5.4	19.2	6.7	6.7	62
Glu–L	9.5	90.5^c^	0.8	4.8	0.6	0.5	94.1
Xyl–L	37.5	62.5^c^	1.1	91.3	2.7	1.9	3.0
Xyl–Glu–L	13.8	86.2^c^	0	58.7	12.1	2.3	26.9
Hardwood (*Betula verrucosa*) [20]							
Glu–Xyl–L	29.2	70.8^c^	0	41.7	11.8	10.0	36.6
Glu–Xyl–L	15.9	84.1^c^	0.9	16.2	0.5	0.7	81.7
Xyl–L–Gal	35.8	64.2^c^	1.0	75.9	2.2	2.7	18.2
Herbaceous plant (*Maize stem*) [83]							
Xyl–L–Ara	38.3	50.3	10.5	83.0	ND	0^7^	6.4
Xyl–L–Glu	18.7	70.7	5.9	82.6	ND	0^7^	11.5
Herbaceous plant (*Sisal*) [85]							
Glu–L	7.8	92.2^c^	1.5	9.0	0.9	0.2	88.4
Xyl–L	24.1	75.9^c^	0.6	89.4	2.6	0.3	7.1
Herbaceous plant (*Abaca*) [85]							
Glu–L	4.4	95.6^c^	0.3	4.1	0.5	0.1	95.0
Xyl–L	29.4	70.6^c^	3.4	75.5	13.0	0.3	7.8
Not fractionated LCC from herbaceous plants							
LCC-AcOH (Bamboo) [25]	19.9	48	3.8	32.9	3.1	1.0	59.2
Björkman LCC (A. donax) [24]	34.8	65.2	6.2	59.2	0.1	3.3	28.9
Björkman LCC (Wheat straw) [23]	16.03	75.1^a^	9.3^a^	75.1^a^	2.2^a^	0^a^	13.4^a^
LCC-WE (Rice straw) [82]	27.7	63.9	13	80.1	0.4	2.3	13

^a^Converted to % based on the data presented in the source

^b^12/24 h of ball milling

^c^Calculated by authors by subtraction the portion of lignin (%) presented in the LCC fraction from 100%

Another study reports that Xyl–L and Glu–L fractions isolated from spruce wood have condensed and linear structures, respectively [17]. Oinonen et al. [78] hypothesize random crosslinks between galactoglucomannans, xylans, and lignin in Norway spruce. It is reported that the glucomannan–lignin fraction is water soluble, which suggests a low crosslinking degree in this complex, since extensively cross-linked polymers are typically insoluble in water [53]. Takahashi and Koshijima [69] also assume that softwood LCC consists of small and repeating lignin units bound to the polysaccharide chain.

## Hardwood LCC structure

Dammstrom et al. [22] propose that xylan in hardwood presents in three forms, one of which is glucuronoxylan attached to cellulose units, the second form xylan is a part of xylan–lignin complexes, and the third one is free xylan. Koshijima and Watanabe [53] state that xylose is the main sugar for benzyl ether linkages. In other research, it is reported that 50% of glucan moieties are involved in benzyl ether linkages with 5–10% xylan contribution [18]. Takahashi and Koshijima [68] state that lignin and glucuronoxylan are linked by benzyl ester bonds with 30% uronic acid. Takahashi and Koshijima [69] propose hardwood LCC structure in the form of a very long polysaccharide chain linked to a few large lignin moieties.

## Non-wood LCC structure

The LCC of grasses mainly includes arabinoxylans bridged to lignin via ferulate esters [62, 79]. Arabinoxylans play an important role in LCC linkage formation, due to the conceivable existence of covalent bonding between arabinose, xylan, and lignin moieties in forage crops [80]. In addition, xylan is reported as a main component-binding lignin and carbohydrates in bamboo, rice straw, and ryegrass [72, 81–83]. It is proposed that lignin in wheat straw is bound to glucan and xylan moieties via ferulate acid and PhyGlc linkages, respectively [23, 84]. In another study, You et al. [24] suggest that PhyGlc bonds exist between cellulose and lignin in herbaceous plants due to the abundance of these bonds. However, Rio et al. [85] report that no signs of benzyl ether, ester, or PhyGlc linkages are found in a glucose–lignin fraction of sisal and abaca. It is also proposed that xylan is linked to lignin via PhyGly bonds in bamboo [25]. Other work confirms that PhyGlc is bound to guaiacyl and syringyl lignin units with xylan moieties in abaca and sisal [85].

## LCC in softwood pulp

As stated earlier, LCC can exist in pulp, and it can also be extracted from spent liquor. The analysis of pulp reveals that 85–90% of lignin remains in softwood kraft pulp linked to carbohydrates in LCC forms [30, 86]; whereas, in oxygen-delignified pulp, all lignin units are involved in LCC [86].

Due to the galactoglucomannan decomposition during kraft pulping, LCC is present in kraft pulp as Glu–L, GM–L–Xyl, and Xyl–L–GM fragments with 12, 45, and 27% of total relative amounts of lignin found in pulp, respectively [19]. It is apparent that hemicellulose–lignin complexes contain both xylan and glucomannan in different proportions, implying that lignin crosslinks with xylan and glucomannan in softwood kraft pulp [30]. Tenkanen et al. [29] reported that the degradation of xylan during the enzymatic hydrolysis of pine kraft pulp significantly enhances the decomposition of glucose, while the hydrolysis of glucomannan does not improve the decomposition of cellulose in the same manner as that of xylan. In addition, it is found that the hydrolysis of glucomannan increases after a considerable removal of xylan. These results imply that xylan is partially covered by glucomannan, whereas, in pine kraft pulp, xylan is entrapped by glucose [29]. The increment in the degree of oxygen delignification leads to the degradation of Glu–L complexes, whereas the GM–L–Xyl and Xyl–L fractions obtained from oxygen-delignified softwood pulp included 80% and 20% of total lignin content in pulp, respectively [19, 86]. Therefore, it is proposed that GM–L–Xyl complex is resistant to oxygen delignification, which might be attributed to the high alkali resistance of phenyl glycosidic and benzyl ether bonds present in this complex.

In addition, with the increment of oxygen delignification intensity, the relative amount of xylan in the GM–L–Xyl fraction decreases, while the relative content of lignin in these complexes increases. At the highest severity of oxygen delignification, almost all lignin moieties in pulp have been found to be linked to glucomannan units. This leads to the conclusion that the main issue of oxygen delignification of softwood species is attributed to glucomannan LCC [86].

## LCC in hardwood pulp

LCC fractionation of eucalyptus kraft pulp shows that Glu–Xyl–L and Xyl–L fractions include 8% and 12% of total lignin present in the eucalyptus pulp, respectively. The compositional analysis of LCC fractions present in birch kraft pulp shows that Xyl–L–Glu contains 34% of total lignin and Xyl–L contains 16% of lignin units [20]. Table 3 lists the composition of LCC fractions extracted from softwood and hardwood kraft pulps.

**Table 3:** Composition of LCC fractions from softwood and hardwood kraft pulp

LCC fraction	Relative composition of LCC fraction, %		Relative carbohydrate composition, %				
	Acid-soluble lignin	Total carbohydrates	Ara	Xyl	Man	Gal	Glu
Softwood Kraft pulp (*Spruce*) [19]							
Glu–L	2.4	96.0	0.5^a^	3.6^a^	4.2^a^	0.0^a^	91.7^a^
GM–L–Xyl	35	41.7	2.9^a^	26.7^a^	48.4^a^	4.1^a^	17.8^a^
Xyl–L–GM	23	75.6	5.8^a^	75.1^a^	8.3^a^	0.5^a^	10.2^a^
Oxygen-delignified softwood pulp (*Spruce*) [19]							
GM–L–Xyl	22.8	77.0	1.4^a^	11.5^a^	62.8^a^	4.2^a^	20.1^a^
Xyl–L	5.1	97.9	2.5^a^	20.2^a^	58.8^a^	3.3^a^	15.3^a^
Hardwood kraft pulp (*Eucalyptus globules*) [20]							
Glu–Xyl–L	0.5	99.5^b^	0.7	10.6	0.3	0.1	88.4
Xyl–Glu–L	0.83	99.2^b^	0.4	92.4	0.9	0.7	5.6
Glu–L	1.48	98.5^b^	1.5	0.8	0.2	0.2	97.3
Hardwood kraft pulp (*Betula verrucosa*) [20]							
Xyl–Glu–L	1.2	98.8^b^	0.2	86.9	0	0	12.9
Xyl–L	0.33	99.7^b^	0	97.7	1.2	0	1.1
Glu–L	0.6	99.4^b^	0.5	0.6	0.2	0	98.8

^a^Converted to % based on the data presented in the source

^b^Calculated by authors by subtraction the portion of lignin (%) presented in the LCC fraction from 100%

## LCC in spent liquors

It is suggested that lignin–carbohydrate complexes can be extracted and dissolved along with lignin and hemicelluloses in the spent liquors of biomass pretreatment [19, 87]. Tamminen et al. [88] proposed the existence of xylan–lignin and galactan–lignin complexes in the spent pulping liquor (black liquor) of the kraft process. It is proposed that some of xylan and glucomannan moieties are dissolved in black liquor along with lignin [29].

In another study, Fatehi et al. [89] propose LCC’s presence both in prehydrolysis liquor (PHL) generated in pretreatment of hardwood chips with saturated steam and in the spent liquor (SL) of neutral sulfite semichemical pulping (NSSC) process, in which hardwood biomass is treated with sodium sulphite and carbonate. In addition, Tarasov et al. [42] reported the existence of LCC in the hydrolysate obtained via flow-through autohydrolysis of softwood chips. It was found that the hydrolysate produced with a high liquid-to-solid (L/S 10/1 wt./wt.) ratio contains 19% of lignin in the LCC form. Furthermore, 89% of lignin moieties bound to carbohydrates in the hydrolysate are generated under a high temperature and lower L/S ratio [90].

## LCC properties

The MW of LCC is not widely discussed in the literature. Table 4 tabulates the properties of LCCs extracted from various sources. The MW of LCC prepared from poplar is reported to range from 9800 to 17,500 g/mol [91]. LCC extracted from non-wood species features a very wide range of molecular weights. The MW of LCC from *Prunella vulgaris* is estimated to be 8500 g/mol [92], whereas the MW of wheat straw LCC is around 38,700 g/mol [23].

**Table 4:** LCC properties

LCC source	Mw, g/mol	Thermal properties [94, 95, 99]			Elemental analysis, % [95, 102]				
		T_onset_, °C	Degradation at 590 °C, wt %	T_g_, °C,	C	H	N	S	O
Hardwood	9800–17,600 [91]	ND	ND	ND	51.9	5.8	ND	ND	42.4
Softwood	12,000 [101]	220–260	45.7	ND	59.6	6.3	1.1	2.2	ND
Non-wood	8500–38,700 [23, 92]	277	55.6	166	62.8	5.4	ND	ND	ND

The molecular weights of xylan (Xyl)- and glucan (Glu)-rich LCC complexes obtained from softwood kraft pulp are estimated to be in the range of 8000–35,000 g/mol and 15,000–45,000 g/mol, respectively [86]. It is reported that the MW of lignin and hemicelluloses present in these LCC fractions is related to the relative lignin content of kraft pulp. The Xyl–L fraction obtained from pulp with lower lignin content possesses lignin and hemicelluloses with a higher MW [86]. In addition, hardwood PHL and SL of NSSC process are reported to contain LCC with the molecular weights of 2000 and 1500 g/mol, respectively [89].

The thermal stability of LCC is affected by various factors, such as interunit structures, functional groups, degree of condensation, and molecular weights [93]. Experimental conditions also impact LCC’s thermal stability. In one study, Nassar and MacKay [94] report that LCC from spruce starts to decompose in the temperature range from 220 to 260 °C, whereas the degradation onset temperature (*T*_onset_) of spruce lignin is reported to be 210–220 °C. This difference could be related to the hemicellulose presence in LCC, as hemicelluloses contain more inherent moisture than does lignin [94]. Another study states that softwood LCC possessed *T*_onset_ of 236 °C, whereas LCC and carbohydrate-free lignin from bagasse started to decompose at 277 and 268 °C, respectively [95]. At 590 °C, the weight of softwood LCC decreases by 45.7%, whereas LCC isolates from bagasse and carbohydrate-free lignin experiences 55.6 and 52.5% weight loss, respectively [95]. A lower degradation of softwood LCC could be related to their bonding extent, which decomposes slowly at elevated temperatures [95]. Molecular weight, molecular weight distribution, and degree of crosslinking impact T_g_ value of LCC [96]. In the past, lignin was reported to have *T*_g_ between 80 and 193 °C [97], while hemicellulose and cellulose had *T*_g_ in the range of 200–250 °C and 150–220 °C, respectively [98]. The *T*_g_ of LCC from bamboo is reported to be 166 °C [99].

The elemental analysis of softwood and bagasse LCC is reported by Singh et al. [95]. Carbon and hydrogen contents of both LCCs are estimated as 63–60% and 5.4–6.3%, respectively. These values are similar to carbohydrate-free bagasse and softwood kraft lignin [95, 100].

Another important parameter of LCC is anti-UV activity. The anti-UV activity is determined by selective index (SI) [103, 104]. SI is defined as the ratio of two parameters: 50% cytotoxic concentration (CC_50_), which is defined as the amount of the compound (μg/mL) required for the reduction of the number of living cells by 50%, and 50% effective concentration (EC_50_) that raises the viability of UV-irradiated cells by 50% [105, 106]. In other words, SI quantitatively expresses the ability of the compound (LCC) to defend cells from UV-induced damage [104].

It is reported that the LCC produced from pine cone and pine seed shell extracts shows anti-UV activity with SI of 24.8–38.1 and 25.6, respectively. A similar anti-UV activity (SI = 38.5) is reported for herbaceous LCC (Sasa senanensis Rehder leaves), whereas the lignin extracted via alkali treatment shows a significantly higher anti-UV activity with an SI of 61.5 [103].

## LCC extraction

Different researchers claim the presence of LCC for altered materials in different studies. In 1935, Hibbert and coworkers proposed the presence of lignin–xylan complexes in spent liquor obtained from extraction of spruce saw meal treated with a mixture of anhydrous ethylene alcohol glycol and hydrogen chloride [107]. In a later study, Merewether [108] reported the presence of xylan–lignin complexes in the spent liquor produced via ethanolysis (in the presence of sodium bicarbonate) of eucalyptus wood meal. In 1953, Traynard [109] and coworkers reported the extraction of LCC from poplar species via water hydrolysis at 140 °C [53]. However, the extraction of LCC via fractionation studied by Björkman systematically for the first time [110, 111].

## Björkman LCC

**Fig. 2:**
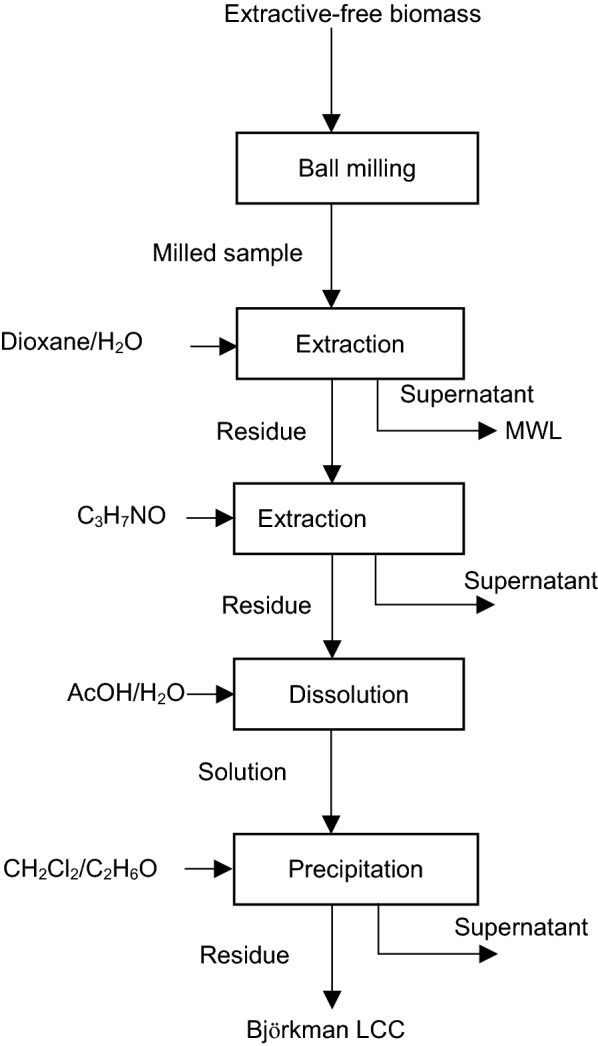
Björkman’s method for LCC preparation [111, 112]

The methods for LCC extractions established by Björkman [110, 111] became a milestone in the investigation of lignin and LCC’s structure and composition. Figure 2 outlines Björkman’s procedure for LCC preparation. In this method, biomass is saturated with toluene prior to milling for 48 h [111, 112]. Afterward, the milled material is mixed with 1,4-dioxone/water (96/4 vol./vol.) solution in a wood/solvent ratio of 1/10 wt/wt and is stirred for 24 h at ambient temperature under a nitrogen atmosphere [112]. Then, the solution is centrifuged. The evaporation of its solvent from supernatants will help separation of milled wood lignin (MWL) [112].

It is reported that MWL represents up to 50% of total lignin content of wood [51, 111]. The precipitates of the centrifugation are then extracted with dimethylformamide (C_3_H_7_NO) or dimethyl sulfoxide ((CH_3_)_2_SO)). The obtained material is purified by dissolution in 50/50 vol/vol acetic acid/water (AcOH/H_2_O) mixture followed by precipitation via washing with a dichloromethane/ethanol (CH_2_Cl_2_/C_2_H_6_O) mixture. The produced precipitates are considered as Björkman LCC. This product is comprised of 16–34 wt% lignin and 66–84 wt% carbohydrates [111, 112].

## LCC extraction via acetic acid (LCC-AcOH)

**Fig. 3:**
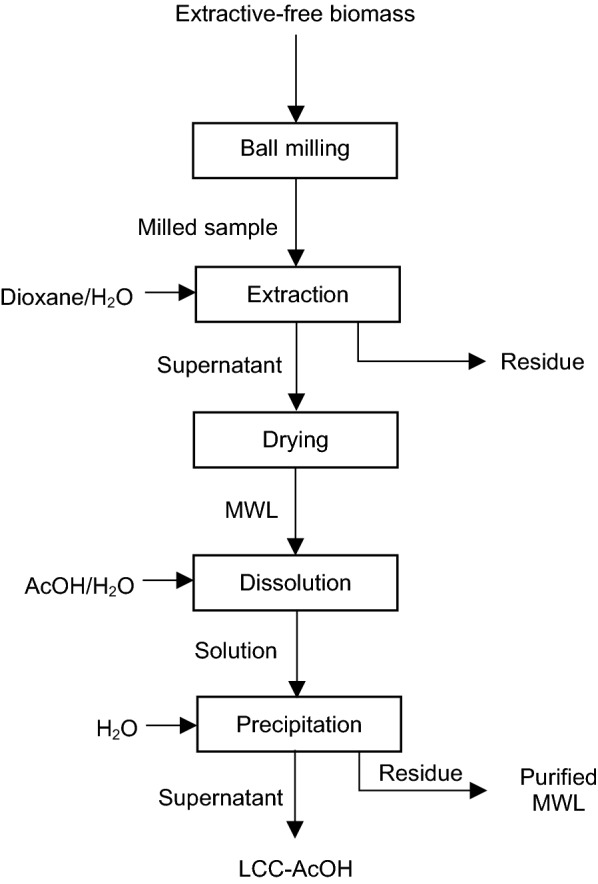
LCC-AcOH preparation procedure [21, 24]

Björkman’s procedure uses solvents with high boiling points, such as dimethylformamide or dimethylsulfoxide [53]. Balakshin et al. [15, 21] and You et al. [24] reported the procedures for LCC extraction from softwood (pine), hardwood (birch), and herbaceous biomass (A. donax). Figure 3 presents the process of LCC preparation using acetic acid (AcOH). In this method, extractive-free wood sawdust is ground by planetary ball milling for 5 h and 600 rpm [21]. In case of herbaceous biomass, the extractive-free material is subjected to planetary ball milling for 12 h and 450 rpm [24]. Then, the produced material is treated by 96/4 vol./vol. 1,4-dioxane/water mixture in accordance with the Björkman’s procedure. Afterward, the solvent is evaporated in vacuum, and then, a few drops of deionized water are added to the solid material to remove traces of dioxane followed by rotary evaporation. The dried material is considered as MWL. Then, MWL is dissolved in 90% aqueous AcOH (at 20 mL/g ratio). The addition of water to the mixture leads to precipitations of purified MWL. Then, the supernatant of this process is collected and lyophilised. Furthermore, the dried material is treated with a few drops of water for AcOH removal. After repeating the purification procedure three times, the dried material is considered to be LCC-AcOH [21, 24].

## LCC extraction with hot and cold water treatment

**Fig. 4:**
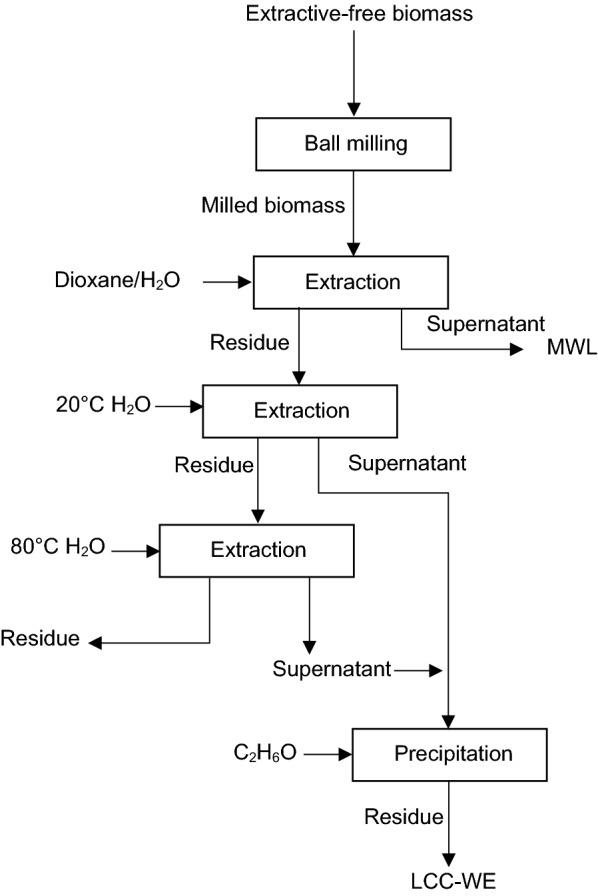
LCC-WE preparation procedure [113]

Another method for LCC extraction was developed by Watanabe [113] and coworkers in 1987. Figure 4 outlines the procedure for LCC-WE preparation. In this method, woody materials are ground, and then, MWL is extracted using an 80/20 vol./vol. dioxane/water solution. The obtained residue is first treated with cold water (20 °C), and washed and then treated again with hot water (at 80 °C). The dissolved materials of these processes are precipitated with ethanol (C_2_H_6_O) and considered as LCC-WE [53, 113].

## LCC fractionation

Softwoods, hardwoods, and non-woods have different morphologies and compositions of lignin and carbohydrates, which results in variations in their LCC properties. LCC extracted from softwood is claimed to have hemicelluloses, such as galactoglucomannan (GGM), glucomannan (GM), arabino-4-*O*-methylglucoronoxylan (Xyl), and arabinogalactan (Gal), bound to lignin (L) moieties [82]. Sugars of LCC in hardwoods consist of 4-*O*-methylglucoronoxylan, whereas LCC from non-woods are composed of arabino-4-*O*-methylglucoronoxylans [82]. LCC fractionation procedures reveal more detailed information regarding the structure and composition of various LCCs, which present in biomass and pulps.

## LCC fractionation via enzymatic hydrolysis and barium hydroxide

**Fig. 5:**
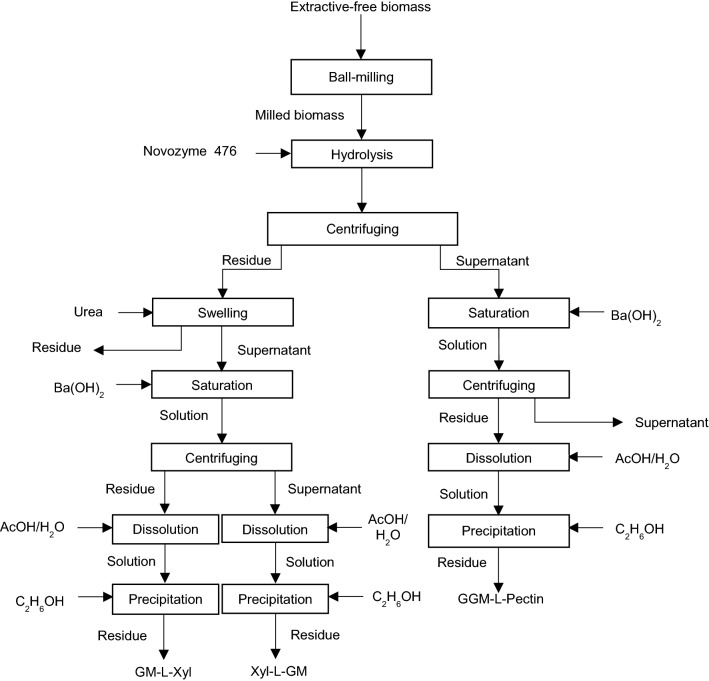
LCC fractionation with combined application of enzymatic hydrolysis and barium hydroxide solution [2, 114]

Lawoko proposes a combination of ball milling, enzymatic hydrolysis, and treatment with barium hydroxide (Ba(OH)_2_) for fractionating LCC [19, 30, 86, 114]. The procedure for LCC fractionation via this method is shown in Fig. 5. First, the extractive-free spruce wood species are ball-milled for 3 h. Next, the milled substance is treated with endoglucanase enzymes (Novozyme 476) followed by centrifugation. Then, the hydrolysate of this enzymatic treatment is treated with 5% aqueous barium hydroxide ((Ba(OH)_2_) for 2 h after centrifugation, which leads to the precipitation of solid material [114, 115]. The generated precipitate is dissolved in 1/1 AcOH/H_2_O solution and then precipitated again via ethanol supplement. After dialysis and drying, the produced material is considered GGM–L–Pectin fraction [2, 114]. The precipitates of enzymatic hydrolysis are swollen in urea for 24–48 h at room temperature. Afterward, the soluble part of the urea mixture is mixed with Ba(OH)_2_, resulting in the formation of two LCC fractions; GM–L–Xyl is found in residue due to its poor solubility, and highly soluble Xyl–L–GM fraction dissolves in supernatant. Next, GM–L–Xyl and Xyl–L–GM fractions are separated by centrifugation. Then, GM–L–Xyl fraction is purified with AcOH/H_2_O solution and re-precipitated in ethanol as described above. Xyl–L–GM portion, remaining in the barium hydroxide solution, is also dissolved in 50% aqueous AcOH solution and precipitated in ethanol. Afterward, the obtained fractions are dialysed and freeze-dried [2, 114].

## LCC fractionation via DMSO/tetrabutylammonium hydroxide (TBAH) mixture

The degradation of β-*O*-4 interunits of syringyl lignin and high solubility of these lignin segments in water after endoglucanase hydrolysis and urea treatment indicates the fact that the procedure should be modified for hardwood LCC preparation [20]. Li et al. [31] proposed a method for fractionation of hardwood LCC, which includes ball milling for 12–24 h and dissolution in 50/50 vol./vol. DMSO/tetrabutylammonium hydroxide (TBAH) mixture. This leads to the dissolution of cellulose components of hardwood, but the lignin structures in LCCs remain intact [31]. Mixing the product with water results in the precipitation of LCC. The lyophilisation of the precipitates generates Glu–L fraction in the precipitates and Xyl–L in the solution.

## LCC fractionation via alkaline extraction and enzymatic hydrolysis

**Fig. 6:**
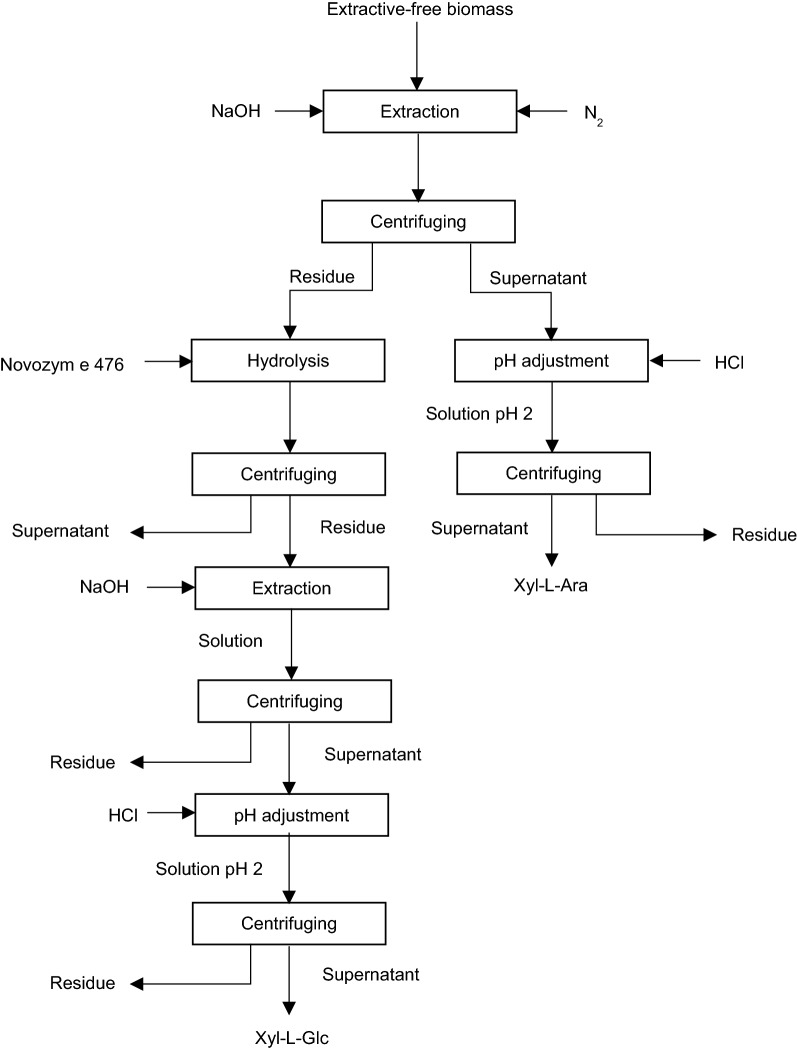
Procedure for isolation of alkali-soluble LCC fractions [83]

Sipponen et al. [83] reported an efficient method for the isolation of alkali-soluble LCC fractions from non-wood plants (Maize stem) via combined application of alkaline extraction and enzymatic hydrolysis. Figure 6 depicts the procedure for alkali-soluble LCC isolation. This procedure describes the extraction of extractive-free biomass with 0.5 M sodium hydroxide (NaOH) solution for 24 h at room temperature under nitrogen (N_2_) atmosphere. This treatment leads to the formation of precipitates and dissolved substances. The product is centrifuged, and the generated residue and supernatant are separated. Then, the pH level of the obtained supernatant is adjusted to 2 by hydrochloric acid (HCl) supplement. The acidified solution is kept for 16 h in the dark and then centrifuged for collection of the insoluble part. The generated suspension is lyophilised, and the obtained solid material is considered as Xyl–L–Ara complex. The precipitate produced from the initial NaOH solutions is treated with Novozyme 476 enzyme, which leads to the generation of hydrolysed suspension. The suspension is centrifuged, and hydrolysed solids are precipitated, washed, and lyophilised. Then, the dried residue is extracted with 2 M NaOH at ambient temperature under N_2_ atmosphere. Afterward, Xyl–L–Glu complex is isolated from alkaline extract via acidification, as described above [83].

## LCC fractionation via universal method

**Fig. 7:**
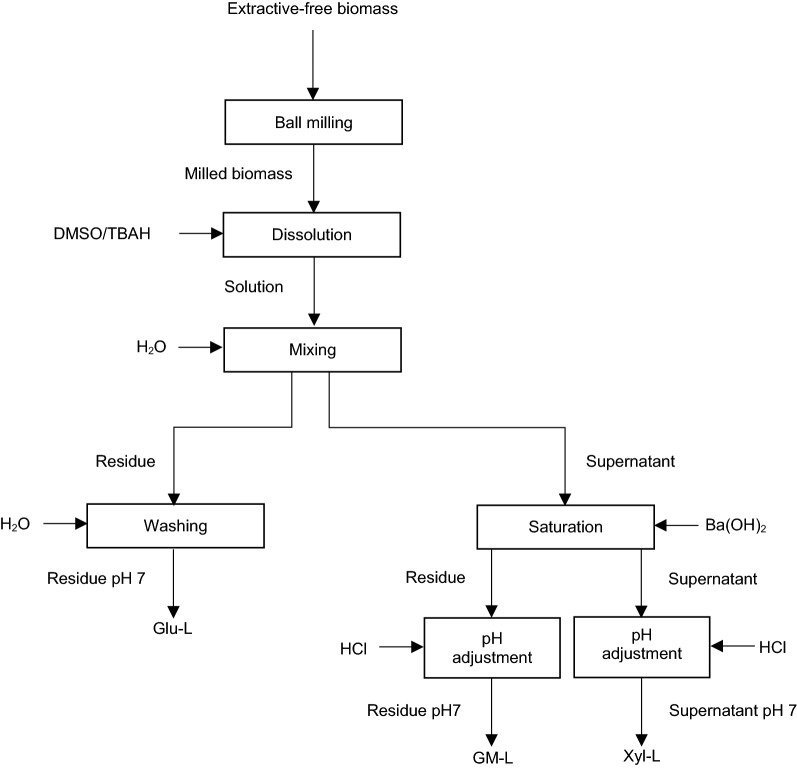
Procedure for universal LCC fractionation [115]

Du et al. [115] reported a universal fractionation process for LCC from lignocellulosic biomass, which combines the procedures developed by Lawoko et al. [19] and Li et al. [31]. Figure 7 depicts the procedure for universal fractionation of LCC. In this method, the extractive-free biomass is first ground by ball milling and then dissolved in DMSO/TBAH solution in accordance with the method developed by Li et al. [31]. It is reported that, after 12 h of ball milling, the milled materials of hardwood, softwood, and herbaceous species entirely dissolve in DMSO/TBAH mixture [31, 115]. The obtained solution is diluted with water, generating two phases of residue and supernatant. The generated residue is washed with water and lyophilised to obtain the Glu–L fraction. The produced supernatant is saturated with Ba(OH)_2_, which leads to the aggregation of barium ions with GM–L fraction and its further precipitation. Both the precipitate and the supernatant are neutralized with HCl, then purified and dried. The materials collected from the precipitate and supernatant are considered as GM–L and Xyl–L fractions of LCC, respectively [115]. It is also reported that the molecular weight of LCC generated in this process is extremely high. Glu–L, GM–L, and Xyl–L fractions have the MW of 490,000 g/mol, 63,000–160,000 g/mol and 18,000 g/mol, respectively [115]. Considering the complete dissolution of the examined wood and herbaceous species in DMSO/TBAH mixture, it is suggested that this method can be applied for the fractionation of LCCs present in pulp and other processed lignocellulosic materials [115].

## Fractionation of pulp LCC

**Fig. 8:**
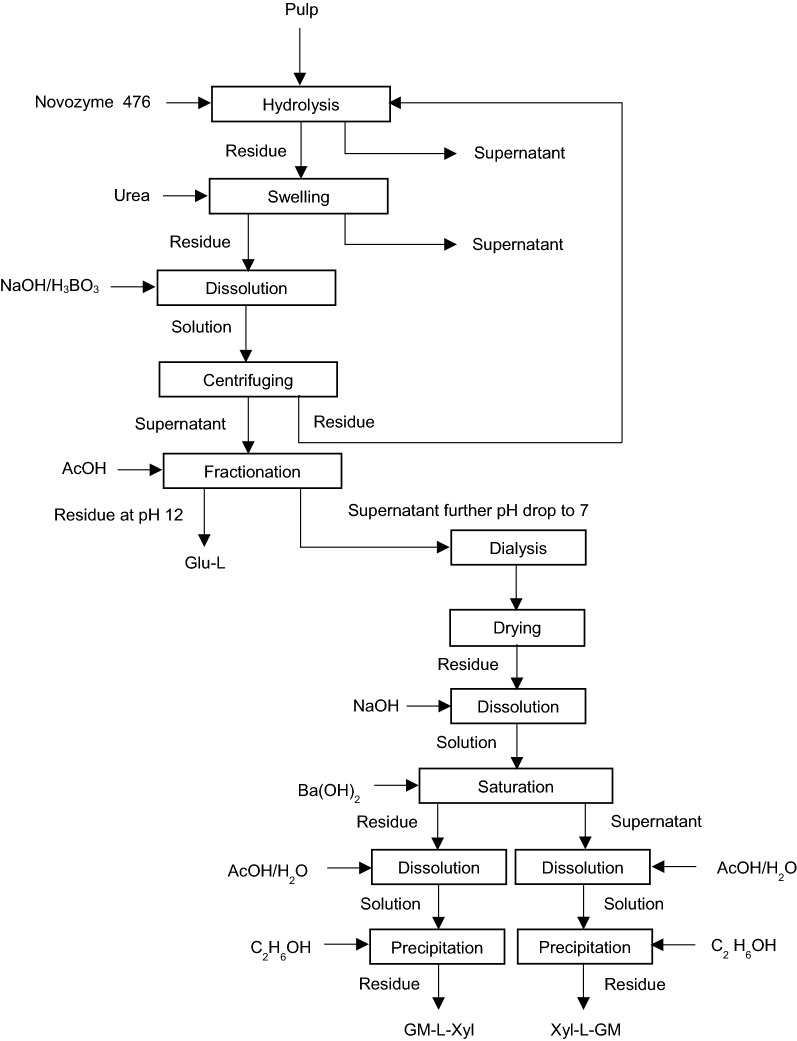
Procedure for pulp LCC fractionation [30, 86]

It is well known that the pulping process leads to a significant delignification of biomass. As stated earlier, benzyl ether and phenyl glycosidic lignin–carbohydrate linkages are alkali-stable, and, hence, will persist through the pulping process [30]. Gierer and Wannstrom [28] suggest the formation of new LCC bonds during the pulping process. In another study, Tenkanen et al. [29] investigated the existence of linkages between lignin and carbohydrates in softwood and hardwood kraft pulp via selective enzymatic hydrolysis of cellulose, xylan, and mannan units. The association between lignin and xylan, glucomannan, and glucose units in pine kraft pulp and lignin–xylan bonding in birch kraft pulp is proposed [29]. To quantify LCC formation in these processes, Lawoko and et al. [30] designed a protocol for fractionation of the LCC obtained from softwood kraft pulp and oxygen-delignified pulp. Figure 8 outlines the protocol for fractionation process of LCC material present in pulp.

In this process, pulp is hydrolysed with endoglucanase (Novozyme 476) enzyme for 48 h. Then, the produced hydrolysate is centrifuged and the precipitated material is separated. The residue is swollen in urea solution overnight at room temperature. Afterward, the material is centrifuged to separate the insoluble part from the supernatant. The insoluble part is then washed with water for the removal of urea and is dissolved in alkaline (18% NaOH) borate (4% H_3_BO_3_) solution for 4 h at ambient temperature for the dissolution of mannose polysaccharides [20]. The precipitate generated via centrifuging of the alkaline borate solution is washed and recycled to enzymatic hydrolysis stage. The supernatant obtained via centrifugation of alkaline borate solution is subjected to pH adjustment via acetic acid supplement causing precipitation of Glu–L fraction at pH 12. Glu–L fraction is separated with centrifugation; where the pH of the solution drops to 7. Since no precipitate is formed at pH 7, the solution is dialyzed and lyophilised, and then, the dried material is dissolved in 0.2 M NaOH. The produced solution is treated with 5% aqueous Ba(OH)_2_, which leads to the precipitation of glucomannan rich material. The obtained fraction is collected and re-dissolved in 0.2 M NaOH and again is precipitated with barium hydroxide solution. After that, the precipitate is mixed with 1/1 AcOH/H_2_O vol./vol. solution and then precipitated via ethanol supplement. After the generated solid material is dialyzed and freeze-dried, it is considered GM–L–Xyl fraction. The suspension obtained after the second treatment with aqueous Ba(OH)_2_ and GM–L–Xyl fraction separation is mixed with 50% aqueous AcOH solution and then precipitated as described above. After the produced substance is purified, it is considered Xyl–L–GM portion. The application of this procedure for fractionation of LCC present in oxygen-dignified softwood pulp results in Xyl–L–GM fraction [30, 86].

The method established by Björkman is a widely used procedure for LCC extraction. However, this technique is time-consuming and involves the usage of solvents with a high boiling point. The application of AcOH or dioxane significantly reduces the time required for LCC extraction. LCC fractionation with enzymatic hydrolysis in combination with Ba(OH)_2_ or alkaline treatment is a suitable method for softwood and herbaceous species. However, syringyl lignin moieties became water soluble after enzymatic hydrolysis, which makes enzymatic treatment inapplicable for fractionation of hardwood LCC. The application of DMSO/TBAH along with Ba(OH)_2_ is considered a universal method for LCC fractionation. However, the LCC fragments obtained after universal LCC fractionation demonstrate extremely high molecular weights, which hinders its analysis with NMR due to its low solubility in solvents required for NMR analysis. For facilitating NMR analysis and molecular weight reduction, the obtained LCC fractions could be hydrolyzed with enzymes, but enzymatic treatment can be expensive and time-consuming. A new or improved method is needed for fractionating LCC more effectively.

## Analysis of LCC

NMR has primarily been used for analyzing the structure of LCC. However, other methods such as alkaline, acidic degradation, oxidation, methylation, and enzymatic analysis are also useful for LCC structure’s analysis [18, 116, 117].

## Ester linkage analysis via alkali degradation of LCC

**Fig. 9:**
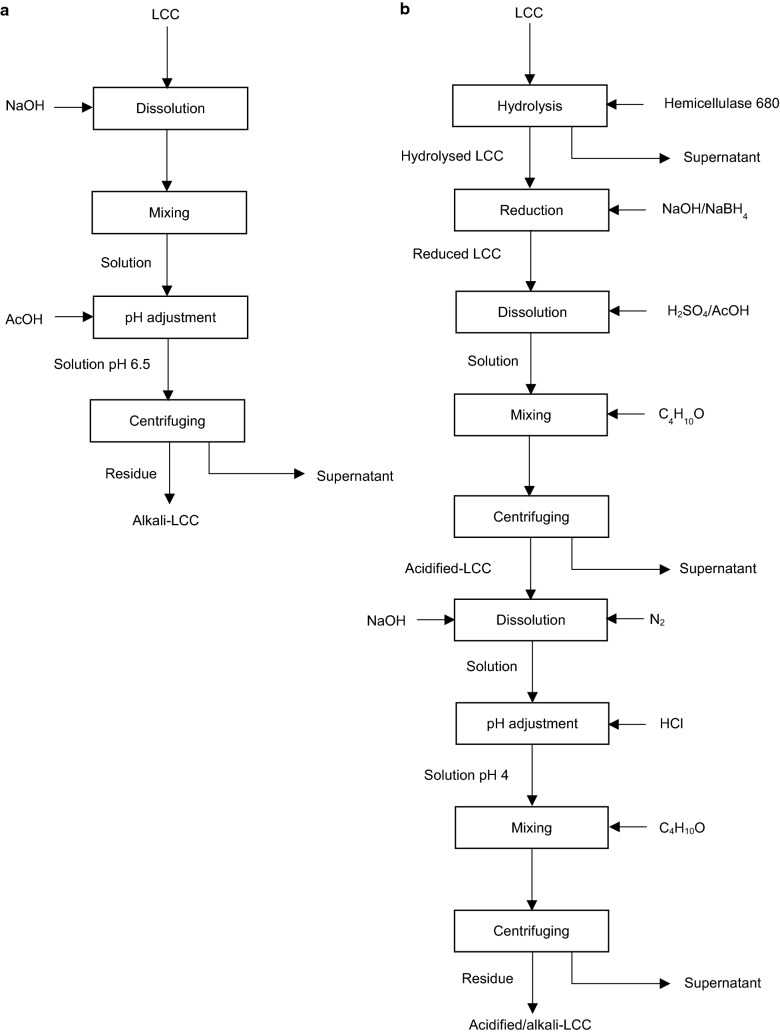
**a** Alkali-LCC [68, 118] and **b** acid/alkali-LCC [55, 120] degradation procedure

Alkali degradation is widely applied for ester bond identification in hardwood [68] and softwood LCC [118]. The analysis is based on ester bond saponification, lignin, and polysaccharide disassociation [118, 119]. The procedure for alkali degradation of LCC is presented in Fig. 9a. According to this methodology, LCC is dissolved in sodium hydroxide (0.1 M NaOH) solution for 1.5–2 h at room temperature [68, 118]. The solution is then neutralized with AcOH to pH 6.5 and centrifuged. The precipitate is washed with water and lyophilised [118]. The comparative IR analysis of untreated LCC and alkali-treated LCC demonstrates the absence of 1730 cm^−1^ in the spectrum of alkali-treated LCC, which confirms the complete saponification of ester bonds in alkali-treated LCC preparation [68, 118]. Obst [118] reported that 10–20% of linkages in LCC are presented in the form of esters in the alkali degradation method.

Moreover, the existence of ester linkages can be estimated via sodium borohydride (NaBH_4_) reduction method [68, 118]. In this method, LCC is dissolved in water with the addition of NaBH_4_ and NaOH [118]. This treatment leads to the reduction of esters to the neutral sugars [68]. In one analysis, the comparison of glucuronic acid concentration before and after borohydride reduction reveals that approximately 30% of linkages in beech LCC are ester-type [68]. However, the linkages degraded due to the alkali treatment are not necessary ester bonds, as benzyl ethers with hydroxyl groups are also alkali-liable [67].

## Ether linkage analysis via acid degradation of LCC

The existence of ether bonds in LCC is investigated via the combined application of sodium borohydride reduction, followed by acid treatment of reduced LCC [55, 120]. This method is based on the analysis of new phenolic and benzyl alcohol hydroxyl groups generated due to the hydrolysis of ether linkages [55, 120]. The procedure for the combined acid and alkali degradation of LCC is shown in Fig. 9b. In this method, enzymatically (enzyme hemicellulase 680) treated spruce LCC is first subjected to the sodium borohydride treatment for the reduction of esters as described above [55, 68]. Then, the reduced LCC is subjected to the selective hydrolysis of linkages between arabinose side chains and xylan (arabinofuranosidic bonds) by dissolution of the material in aqueous mixture of H_2_SO_4_/AcOH 1/1 vol./vol. at 90 °C for 2 h. Hydrolyzed LCC is precipitated from the obtained solution via treating with ethyl ether (C_4_H_10_O) followed by centrifugation. Afterward, the produced residue is washed with ethyl ether and dried [55, 120]. The obtained material of acidified LCC is dissolved in 1 M NaOH under N_2_ conditions at ambient temperature for 40 h [55, 120]. Then, the pH of the obtained solution is adjusted to 4 by supplement of 2 M HCl solution and the material is precipitated as described above [120]. This treatment is supposed to saponify all remaining glucuronic acid ester bonds between glucuroxylan and lignin [55, 68]. It is suggested that xylan units remaining in the LCC after this treatment are bound to lignin by ether bonds to xylose moieties [55, 120].

## Phenyl glycosidic linkage analysis via Smith degradation of LCC

**Fig. 10:**
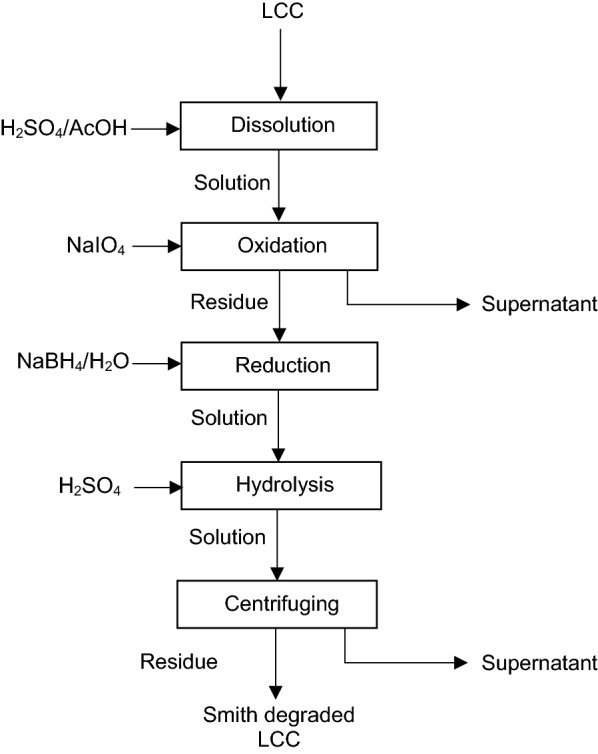
Procedure for Smith degradation of LCC [55, 122]

The glycosidic linkages in softwood LCC are studied via the degradation method developed by Smith and et al. [121]. This method allows the conversion of glycosidic linkages into acyclic acetal bonds, which are liable to acid hydrolysis and can be decomposed via mild acid hydrolysis treatment [55]. The procedure for Smith degradation of LCC is presented in Fig. 10. This method involves periodate oxidation, borohydride reduction, and acid hydrolysis stages. It is proposed that sugar moieties, remained in LCC after these treatments, are bound to lignin [55, 122]. The LCC is first dissolved in 1/1 vol./vol. of H_2_SO_4_/AcOH solution at the approximate LCC/solution ratio of 10/1 wt/wt. The solution is then mixed with sodium periodate (NaIO_4_) and kept for 72 h at 5 °C in a dark place. Afterward, the insoluble material generated in the oxidation is collected and subjected to treatment with sodium borohydride (NaBH_4_) in water for 12 h. Then, the treated material is suspended in H_2_O/AcOH mixture and then hydrolyzed with 0.25 M H_2_SO_4_ solution for 8 h at ambient temperature. The precipitates of this process are collected, washed with water, and treated with sulfuric acid at 100 °C for 12 h [55]. In a related work [122], the oxidation of LCC is performed for 220 h. Then, the solution is purified via dialysis and treated with NaBH_4_ for 15 h; after which the solution is treated with H_2_SO_4_ for 15 h followed by centrifugation for precipitate separation. The collected precipitate is washed with water and ethanol, and then dried [122].

Enzymatic treatment is used as the preparation procedure prior to analysis. This method is widely applied for sugar content reduction of wood and non-wood LCC [17, 55, 80, 120]. In addition, glucuronyl and feruloyl esterases enzymes could be applied for benzyl ester and ferulic acid ester bond studies of LCC [123, 124].

## Paper electrophoresis

LCC structure was studied via paper electrophoresis in 1958 by Lindgren [51, 53, 125]. Electrophoresis is the method of separating ionic particles and its migration with certain velocity as a result of the application of the external electric field [126, 127]. In the paper electrophoresis system, two chambers (anode and cathode) are filled with a conductive medium (electrolyte) and connected by paper strip, which is soaked in the electrolyte at the opposite ends [126]. The velocity of migration is determined by the mobility of charged particles and field strength. The electrophoretic mobility depends on charge density, size, and shape of particles [127]. In this analysis, Björkman LCC and MWL isolated from fir wood are pre-colored with Procion dye and then deposited on the glass–fiber paper strips near the anodic side at two different spots [51, 53, 128]. Then, the ends of the paper strips are placed in the 0.05 N sodium hydroxide solution and the current of 1.8 kV is applied for 45 min [51, 53]. In this work, the LCC was separated into two parts: the slower moving part is composed of carbohydrates and the faster part contained both lignin and hemicellulose moieties. LCC spot was found to move slower than the MWL spot, which confirmed the hypothesis that MWL is a product of dissociation of L–C bonds [51, 53].

## Determination of LCC structure via methylation

**Fig. 11:**
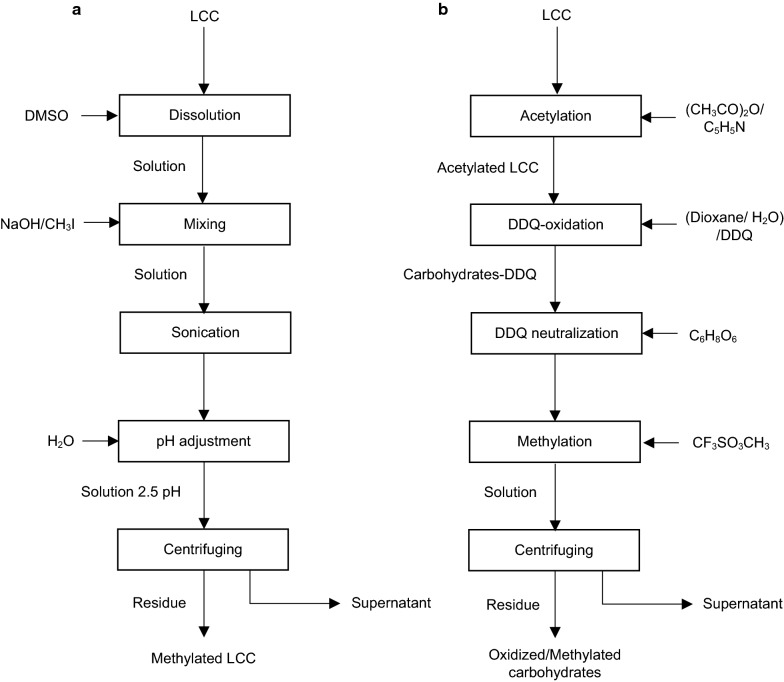
Procedures for LCC analysis by **a** methylation [132] and **b** DDQ/methylation techniques [136]

In the 60s, the methylation analysis was employed as a standard procedure for identifying the chemical structures of oligosaccharides and polysaccharides [129, 130]. This method is based on carbohydrate treatment with methylsulphinyl carbanion and methyl iodide, which leads to the complete hydrolysis of methylated polysaccharides into partially methylated monosaccharides and acetylation of hydroxyl groups [130]. Methylation analysis could be employed as an alternative to NMR spectroscopy for structural analysis of poorly soluble carbohydrates and LCCs [69, 131, 132]. Methylation is also used for studying lignin–carbohydrate linkages [69, 131–133]. The protocol for methylation of LCC of biomass or pulp is presented in Fig. 11a. LCC produced is methylated in accordance with the procedure described by Hakomori [134]. In this method, LCC is dissolved in dimethyl sulphoxide (DMSO) in a nitrogen atmosphere. Afterward, a mixture of sodium hydride (NaH) and methyl iodide (CH_3_I) is added to the solutions. Then, the solution is mixed with chloroform and filtered for sodium iodide removal (NaI). Finally, DMSO is removed from the solution via extraction with water and chloroform is evaporated via drying over anhydrous sodium sulfate (Na_2_SO_4_) [134].

The methylation of LCC generated from kraft pulp can be performed via the method developed by Ciucane and Kerek [135]. In this process, LCC is dissolved in DMSO and mixed with NaOH and CH_3_I solution [131, 132]. The mixture is sonicated for 30 min at ambient temperature for carbohydrate suspension. Then, the sample is mixed with water until a pH of 2.5 is reached. The suspension is then centrifuged at 6000*g* for 20 min [132]. The generated precipitate can be lyophilised and considered as methylated LCC. The success of the methylation process is confirmed by reduction of absorption in hydroxyl group region at 3400 cm^−1^ [133] and increment of absorption in methyl group regions, 2930 cm^−1^ in FTIR analysis [132].

After the methylation, an acid methanolysis of the methylated samples is performed [131, 132] to yield methylated monosugars [18]. The solution is then acetylated, and the obtained alditol acetate mixture is subjected to GC/MS and gas–liquid chromatogram (GLC) analysis [18] for the identification of carbohydrates bonding in acetylated LCCs [68]. The nature of carbohydrate bonding is specified in accordance with unmethylated (but acetylated) sites of monosaccharides [18].

## Determination of LCC structure via combined oxidation and methylation

Methylation analysis allows for studying the nature of LCC bonds. The relative amount of carbohydrates and the specification of linkage in sugars involved in benzyl ether can be determined following another approach. Watanabe and coworkers [136] designed a procedure that included methylation and oxidation with of 2,3-dichloro-5,6-dicyanobenzoquinone (DDQ) for identifying the location and frequency of the bonding sites of sugars to lignin. It is also reported that DDQ particularly attacks nonphenolic benzyl ether linkages, but is neutral to glycosidic bonds in LCC [101, 137].

Figure 11b outlines the procedure of combined application of methylation and DDQ oxidation for the LCC analysis. In this process, methylation is conducted with methyl trifluoromethanesulphonate (CF_3_SO_3_CH_3_) [138] along with the cleavage of ether linkages by DDQ oxidation [136, 139]. The softwood LCC is first acetylated with acetic anhydride ((CH_3_CO)_2_O)) and pyridine (C_5_H_5_N) at 40 °C for 18 h for hydroxyl group protection. Then, the acetylated material is treated in solution of 50% aqueous dioxane and 50% of DDQ at 40 °C for 24 h. This treatment does not affect the acetyl group and glycosidic bonds between sugars, but it separates benzyl ethers from electron-donative benzene skeleton due to oxidation. In this analysis, carbohydrates bound to lignin by ether and ester linkages at the α- and γ-conjugated positions are released due to disruption of lignin–carbohydrate linkages caused by DDQ oxidation [15]. The new hydroxyl groups of carbohydrates liberated from the cleaved LCC bonds are subjected to a methylation procedure for further analysis. For this purpose, the effect of DDQ oxidation is terminated by ascorbic acid (C_6_H_8_O_6_) supplement. Then, the obtained carbohydrates are methylated, in accordance with Prehm [138] by CF_3_SO_3_CH_3_ at 50 °C for 3 h, and methylated carbohydrates are recovered via centrifugation [138]. Afterward, the obtained methylated samples are hydrolysed, reduced with sodium borohydride, and acetylated [136, 138]. This product contains partially methylated alditol acetates, which are subjected to the GC/MS analysis [136]. The position of methoxyl group specifies the location of monosaccharide bonding to lignin [18]. Monosaccharides are indicated in accordance with their retention times by mass spectrometry [136, 138]. Thus, a combined application of methylation and DDQ oxidation allows for the identification of positions and types of carbohydrate bonds to lignin in LCC by α- and γ-ether and ester linkages [68, 136].

## Determination of LCC presence with gel permeation chromatography (GPC) analysis

In other work, the existence of LCC is identified with gel permeation chromatography (GPC) via applying the triple detection technique [87, 89, 90]. The molecular weight of lignin is estimated via UV detector at 280 nm wavelength; the MW of carbohydrates is analyzed using reflective index (RI) and intrinsic-differential pressure (IV-DP) detectors [89]. Similar retention times obtained for UV, IR, and IV-DP pulses suggest the interlinkages between lignin and hemicelluloses, and, hence, the presence of LCC [87, 89]. This method is employed for the investigation of LCC presence in SL of NSSC process, PHL, auto hydrolysis liquor, and hydrothermally treated biomass [87, 89, 90]. In addition, the application of this method allows the estimation of the MW of LCC and carbohydrate-free lignin [89, 90].

However, the GPC analysis does not disclose any specific perception about the nature and amount of lignin–carbohydrate linkages present in the analyzed materials. The presence of ester, ether, and phenyl glycosidic linkages and approximate quantification of these bonds could be determined via alkali, acid, and Smith degradation methods, respectively. In addition, the structure of LCC fractions and location of lignin–carbohydrate bonds could be estimated by methylation or combined with 2,3-dichloro-5,6-dicyanobenzoquinone oxidation/methylation treatment via gas chromatography–mass spectrometry and gas–liquid chromatography analyses. The main drawback of these methods, in comparison with NMR technology, is the complexity of sample preparation.

## Application of NMR technology for LCC analysis

The nuclear magnetic resonance spectroscopy (NMR) technology is widely employed for analysis of the chemical structure and composition of biomass [76, 140, 141]. The main principle of NMR is based on the fact that all atomic nuclei are electrically charged and many of them have a spin moment, which is the result of unpaired spins of the protons and neutrons generating a nuclear magnetic moment [142]. An evaluation of LCC structure and composition via NMR technology is a major landmark in the LCC analysis [18]. Different LCCs have different solubility in various solvents; some LCCs could be dissolved in deuterium oxide (D_2_O), deuterated dimethyl sulfoxide (DMSO-d6) or 50/50 vol./vol. D_2_O/THF-d8 solutions, but some are insoluble in organic solvents, such as tetrahydrofuran-d8 (THF-d8) and chloroform [143]. In general, the high solubility of LCC in DMSO-d6 makes the application of this solvent attractive for the NMR analysis [24].

^1^H NMR, ^13^C NMR, and 2D NMR are widely used for lignin and hemicelluloses analysis [76, 140, 141]. Recently, due to the superimposition of ^1^H and ^13^C frequencies of LCC linkages with other signals of carbohydrates and lignin, the two-dimensional heteronuclear single quantum coherence (HSQC) ^1^H–^13^C NMR technology has been applied for the structural analysis of LCC bonds [21, 144]. The main advantage of two-dimensional technology over one-dimensional technology is its affinity to avoid the overlapping of signal from ^1^H nucleus by correlating it with signals from ^13^C nucleus [145]. This is critical in LCC bond analysis due to a significantly higher dispersion of lignin and carbohydrates signals [146], which leads to a noticeably improved resolution of NMR spectra [145] and more accurate determination of the structure of LCC [146].

^1^H-NMR is reported to provide information on the presence and compositions of the hydroxyl groups in LCC. In one study, Skurikin [147] suggests the existence of carbohydrate units in lignin extracted from oak wood (via ethanol treatment) based on the higher signal intensity of aliphatic hydroxyl protons in LCC than in other lignin samples [148]. Merewether et al. [102] reported that the amount of aliphatic, phenolic hydroxyl groups and free carboxylic groups in hardwood LCC is 0.6, 0.9, and 0.1 per phenylpropane unit (C9), respectively. The lignin present in hardwood contains around 0.32 phenolic hydroxyl groups per C9 unit [149]. The excess of phenolic groups in LCC could be related to the cleavage of alkali-liable linkages during the LCC formation/extraction [102]. In another study, Kosikova et al. [148] investigated the effect of alkaline and acid hydrolysis on the composition of LCC isolated from beech wood via ^1^H-NMR spectroscopy. It is found that both types of hydrolysis do not affect the structure of insoluble lignin in LCCs as no significant changes in distribution of protons in aromatic region are observed [148]. Prior to the ^1^H-NMR studies, LCC samples could be acetylated [102] for a better resolution in NMR analysis [21, 115]. However, acetylation may cause chemical modifications of lignin, which is undesirable [150]. ^1^H NMR analysis takes only a few minutes [151], but the data obtained during ^1^H-NMR assessment may be indistinct due to signal overlapping [53] originating from the short chemical shift dispersion (δ_H_ 12–0 ppm) of ^1^H NMR spectra [150].

^13^C-NMR is reported to provide information on the composition of lignin or carbohydrate parts of LCC [18, 24] and the positions of lignin–carbohydrate bonds on lignin’s side chains [152]. In one study, lignin–carbohydrate bonds are reported to be located at the C_α_-position of lignin moieties in the LCCs isolated from Ginkgo wood [152]. In another study, ^13^C-NMR analysis of the LCC isolated from oat wheat confirms sugar bonds with lignin at α-position of lignin [153]. The natural abundance of ^13^C isotope is as low as 1.1%, which leads to the losses of magnitude in sensitivity [145]. ^13^C NMR technology has a significantly wider shift range (δ_C_ 0–200 ppm) than ^1^H-NMR spectroscopy. However, in the LCC analysis, intensive signals of carbons present in carbohydrates could impede the accurate designation of signals from carbons present in lignin moieties of LCC [152]. Xie et al. [152] apply a method where LCCs are chemically modified for ^13^C-enrichment of the side chain carbons of lignin. This alteration allows for increasing the intensity of signals produced from LCC linkages [152]. Barakat et al. [153] also observed a peak at 81 ppm of ^13^C-NMR spectrum of non-wood LCC, which is assigned to benzyl ether groups. However, these results are not conclusive, as these signals could also be attributed to aryl glycerol and spiro-dienone substructures of lignin and carbohydrates [15, 154]. The ^13^C NMR analysis of lignin material isolated via ball milling can be used for the structural analysis of LCC [155]. It is reported that phenyl glycosidic, benzyl ether, and ester linkages can be indicated by the clusters at δ_C_ 103–96 ppm, 90–78 ppm, and 65–58 ppm in ^13^C NMR spectra, respectively [155]. However, Balakshin et al. [18] stated that ^13^C-NMR analysis cannot be considered as a dependable method for LCC linkage analysis as its signals overlap the signals from lignin or carbohydrate units. In addition, ^13^C-NMR is a very time-consuming analysis, as it takes more than 24 h to obtain a reliable spectrum [151].

## Qualitative analysis using NMR technology

For the accurate investigation and detection of lignin carbohydrate bonds, a combined analysis of signals from both protons and carbons should be conducted. This analysis can be executed with the use of two-dimensional ^1^H–^13^C NMR technology. Numerous two-dimensional NMR techniques are applied for investigating LCC’s structure and composition, such as heteronuclear multiple bond coherence (HMBC) [15], total correlation spectroscopy (TOCSY) [156], and heteronuclear single quantum coherence (HSQC) [17, 21, 140]. The HSQC technology is the most widely employed method due to its diversity in representing structural features and modifications of lignin and carbohydrate units [151]. The HSQC NMR is reported to provide information on lignin carbohydrate linkages in LCC obtained from various softwoods [17, 21, 73], hardwoods [21, 144], and herbaceous plants [23–25]. Balakshin and colleagues reported the direct detection of phenyl glycosidic linkages in the LCC of eucalyptus [18] and pine [15]. In addition, two-dimensional NMR analysis of pine LCC indicates the presence of benzyl ether linkages [15], which confirms the findings reported by Watanabe et al. [136] via DDQ oxidation technique. The absence of signals from α-ester linkage is reported in two-dimensional NMR studies of LCC [15, 17, 141]. Balakshin et al. [15] observed a significant presence of γ-ester in pine LCC, whereas Yuan et al. [144] reported that the signals of γ-esters on the two-dimensional NMR spectra of poplar LCC are indistinct. The two-dimensional NMR technology is also applied for investigating the presence of γ-ether in LCC linkages. However, the region, where these linkages may be located (δ_C_/δ_H_ 65–75/3.0–4.5 ppm), extensively overlapped other areas of the spectrum [18]. Three-dimensional (3D) NMR technology could be employed for deeper investigation of lignin and carbohydrates structural features [18, 157]. 3D HSQC-TOCSY technology provides a combined analysis of ^1^H–^1^H and ^1^H–^13^C correlations. HSQC spectroscopy demonstrates the interconnectivity of protons and carbons spectroscopy, while TOCSY projection associates these with other hydrogen nuclei of the ^1^H–^1^H spin system [158]. Thus, the three-dimensional NMR analysis allows collection of more accurate information regarding certain structural features of lignin and carbohydrates. However, long experimental period (24–48 h) [145] and ability to collect the majority of structural data of lignin and carbohydrates via one-dimensional and two-dimensional NMR spectroscopies impede the application of three-dimensional NMR technology [151].

Application of the latest advances in NMR technology, such as inverse detection and CryoProbes, significantly increases the sensitivity and reduces the duration of experimental periods [145, 159]. Inverse detection technology reduces the time required for 2D ^13^C–^1^H significantly [145] and increases sensitivity of the analysis [160]. CryoProbes increases sensitivity by a factor of 4 in compassion with the standard probes [161]. This would result in reducing the experimental duration possibly by 16-fold and the required concentration of materials by fourfold [145, 161]. Recently, Nishimura et al. [162] employed the 2D HSQC-TOCSY and 3D TOCSY-HSQC CryoProbe technologies for identifying the α-ether linkage existing between mannose in glucomannan and lignin in the LCCs of Japanese red pine. Ralph and Landucci [145] suggest that experiments in inverse detection mode allow studying the materials with a small concentration (≈ 1%).

The concentration of LCC used in 2-D HSQC NMR analysis seems to be different in altered experiments. Yuan et al. [144] solubilized 90 mg of Björkman LCC sample in 0.5 mL of DMSO-d6, whereas Du et al. [17] dissolved 20 mg of enzymatically treated LCC in 0.75 mL of DMSO-d6. Table 5 lists two-dimensional HSQC NMR signal assignments for LCC linkages with DMSO-d6 used as a solvent.

**Table 5:** Two-dimensional HSQC NMR shift in DMSO-d6 for LCC linkages

LCC linkage	δ_C_/δ_H_, ppm
Benzyl ether	
C1-α lignin-C-6 of Glu, Gal, Man, and C-5 of Ara	80–81/4.5–4.7 [160]
C2-α lignin-Xyl	80–81/5.1–4.7 [160]
C2–α lignin-Xyl	81.2/5.1 [21]
Ester	
α-Ester	75/6.1 [160]
γ-Esters	65–62/4.0–4.5 [21, 160]
Phenyl glycosidic	
PhyGlc_1_	100.2/5.03 [17]
PhyGlc_2_	100.3/4.85 [17]
PhyGlc_3_	101.9/4.86 [17]

## Quantitative analysis of LCC linkages using NMR

A quantitative evaluation of LCC linkages in NMR analysis became possible with a method developed by Zhang and Gellerstedt [92]. This procedure involves a combined application of ^13^C and HSQC NMR technologies. The main aspect of this approach is the use of certain clusters of ^13^C spectra as internal references for conversion of the corresponding signals which present in two-dimensional spectra into absolute values [18, 144]. The region between 102 and 162 ppm in ^13^C NMR spectrum is considered as reference, since the peaks belong to 6 aromatic carbon rings and 0.12 vinylic carbons [76]. To obtain a number of substructures present in the region of interest per 1 aromatic unit, the integral areas of these peaks should be divided by 6.12 [76]. Integration of clusters at 103.6–96.0 ppm, 90.0–78.0 ppm, and 64.5–58.5 ppm in a ^13^C NMR spectrum should be applied for the quantitative analysis of PhyGlc, BE and ester linkages, respectively [21, 144]. Amounts of PhyGlc, BE, and ester linkages in Björkman LCC [144] and LCC-AcOH [21] per 100 Ar can be estimated in accordance with the following equations:

1 $${\text{PhyGlc}} = \frac{{2{\text{D}}_{\text{PhGlc}} }}{{2{\text{D}}_{103 - 96/5.5 - 3.8} }} \times \frac{{13{\text{C}}_{103 - 96} }}{{13{\text{C}}_{163 - 106} }} \times 600$$

2 $${\text{BE}} = \frac{{2{\text{D}}_{\text{BE}} }}{{2{\text{D}}_{90 - 78/5.7 - 3.0} }} \times \frac{{13{\text{C}}_{90 - 78} }}{{13{\text{C}}_{163 - 106} }} \times 600$$

3 $${\text{Ester}} = \frac{{2{\text{D}}_{\text{Est}} }}{{2{\text{D}}_{65 - 85/5.0 - 2.5} }} \times \frac{{13{\text{C}}_{65 - 58} }}{{13{\text{C}}_{163 - 106} }} \times 600,$$

where, 2D_PhyGlc_, 2D_BE_, and 2D_Est_ are the volumes of the signals assigned to the PhyGlc, BE, and ester linkages, respectively (Table 5); 2D_103–96/5.5–3.8_, 2D_90–78/5.7–3.0_, and 2D_65–85/5.0–2.5_ are the total resonance of signals in the corresponding areas of the 2*D* spectra; ^13^C_103–96_, ^13^C_90–78_, and ^13^C_65–58_ are the volume of specified clusters signals in the ^13^C spectra and 600 (or 612) is the number of aromatic carbons in 100 monomeric lignin moieties [21].

Another strategy for LCC linkages and lignin interunit quantification is to use the data from HSQC spectra and aromatic units (C_9_) as an internal standard [156]. This approach uses the specific clusters of signals in HSQC spectra, which include all aromatic units. The total amount of C_9_ units in softwood, hardwood, and non-wood species can be quantified by integration values, in accordance with Eqs. 4, 5, and 6, respectively [160, 163]:

4 $${\text{IC}}_{9} = {\text{G}}_{2}$$

5 $${\text{IC}}_{9} = 0.5{\text{IS}}_{2,6} + {\text{G}}_{2}$$

6 $${\text{IC}}_{9} = 0.5{\text{IS}}_{2,6} + {\text{G}}_{2} + 0.5{\text{IH}}_{2,6} ,$$

where IG_2_ is the integration value of cluster assigned to guaiacol lignin (G_2_) units in HSQC spectra [163]. The correlations of the C_2_–C_6_ position of syringyl units (S_2,6_) are twice the amount of syringyl units (S-type lignin); hence, to prevent an overestimation of C_9_ units, half of S_2,6_ integration value (IS_2,6_) was used for C_9_ quantification [163]. Aromatic units in herbaceous species, in addition to G and S units, include hydroxyphenyl units (H-type lignin). Therefore, for the quantification of C_9_ units that present in grasses, half of the integral value of the clusters assigned to C_2_–C_6_ hydroxyphenyl moieties (H_2,6_) is also included [164]. Then, the amount of LCC linkages per 100 Ar could be estimated by Eq. 7 [25]:

7 $${\text{AX}} = \frac{\text{IX}}{{{\text{IC}}_{9} }} \times 100,$$

where AX is the amount of LCC linkages per 100 Ar; IX is the integration value of the objective bond. Zhang et al. [25] applied this method for the estimation of LCC linkages in LCC-AcOH preparation from bamboo.

Another method for the evaluation of LCC linkages involves the integration of a corresponding region of two-dimensional spectra assuming the sum of identified substructures to be 100% [165]. The main disadvantage of this method is its inability to evaluate LCC linkages and other substructures in absolute values [21]. The combined application of data from ^13^C and HSQC NMR provides more reliable information about LCC linkages due to more accurate quantification of aromatic units with ^13^C NMR spectroscopy.

The ^1^H-NMR spectroscopy facilitates the analysis of phenolic hydroxyl groups’ abundance before and after alkali or acid treatment of LCC preparations, which demonstrates the presence of benzyl ester or phenyl glycosidic linkages, respectively. The ^13^C-NMR technology could be also employed for L–C linkages analysis. However, due to the overlapping of carbon signals of lignin, carbohydrates, and L–C bonds, the LCC preparation method for NMR analysis should be modified via chemical treatment [152]. This procedure increases the intensity of signals from the carbons in L–C bonds in the ^13^C-NMR analysis and allows for the identification of LCC structure. On the other hand, the obtained results are not completely reliable as signals arising from the L–C linkages can be obstructed by other lignin-related signals. The application of 2D NMR technology prevents signals’ overlapping, and the accurate quantification of LCC linkages via 2D and ^13^C NMR spectroscopies is one promising method to analyze the structure of LCC. For more detailed investigation of LCC structures, such as γ-ether bonds, 3D HSQC-TOCSY spectroscopy was reported to be an effective method. This technology uses ^1^H–^1^H and ^1^H–^13^C NMR analysis, which leads to more detailed spectra of the heavily overlapped region where γ-ether linkages may be located. In addition, the application of CryoProbes in an inverse detection mode could elucidate the structure and composition of LCCs with very low lignin concentration requirement.

## LCC application

### Anti-microbial and anti-HIV effects of LCC

LCC isolated from *Lentinus edodes* mycelia, pinecone, and pine nut shells via alkaline extraction and acid precipitation show a high anti-UV effect, which could be applied for manufacturing sunscreens [166]. It is articulated that LCC from *Sasa senanesis* Rehder leaves have a higher anti-UV activity comparing with natural polyphenols [104].

In addition, different medicobiologic applications of LCC are suggested [167–169]. Zhang et al. [167] note a high anti-herpes activity of LCC of pine cone and Prunella plant. Sakagami et al. [168] reported anti-HIV, anti-influenza virus, and anti-herpes effects of LCC extracted from pine cones. The antiviral and immunostimulatory effects of LCC from herbaceous plant (*P. anisum*) are also reported by Lee et al. [169]. It is assumed that the mechanism of anti-HIV activity is related to the ability of LCC to inhibit the HIV adsorption and penetration into cells [168]. It is found that the lignin units are more important for anti-HIV activity than sugar moieties. However, the application of phenylpropanoid monomers does not demonstrate any anti-HIV activity, which implies the significance of highly polymerized structure of LCC [168]. Different LCCs from softwood cone or seed shells demonstrate the ability to stimulate anti-microbial activity. It is reported that the anti-microbial activity of LCC is considerably reduced with the carbohydrate unit degradation, which indicates the importance of sugar units for anti-microbial activity of LCC [168].

### LCC-based biological carriers

Lignin–carbohydrate complexes demonstrate good biological compatibility and mechanical resistance [170, 171]. These properties are attributed to a combination of water-repellent, inelastic lignin units, and hydrophilic, flexible carbohydrates in LCCs [91]. Zhao et al. [91, 172] applied LCC fractions from poplar wood for the preparation of spherical biocarriers. Biocarriers are inactive compounds able to attract, keep, and biomagnify certain microorganisms [173]. Zhao et al. [91, 172] estimated the proliferation of liver cells after application of biocarriers prepared from various species of hardwood (Poplar) and softwood (*Ginkgo biloba* L.). Galactose units of LCC are able to recognize liver cells due to the presence of asiaglycoprotein receptors (ASGPR) on hepatocytes, as galactose functions as ligand and bind hepatic cells with these receptors. LCC–hepatocyte complexes are proposed to be able to culture hepatocytes due to the interaction of galactose and ASGPR [172]. The effect of LCC biocarriers on metabolic activity of hepatocytes is evaluated; the results demonstrate the improvement of liver cells proliferation. It is reported that the cell number of human hepatocytes cultured in hardwood LCC carriers and control groups (without application of LCC biocarriers) in a definite time period is 1.84–1.68 × 10^5^ cells/mL and 1.32 × 10^5^ cells/mL, respectively [91]. The implementation of softwood LCC biocarriers increases the number of cells cultured to 6.5 × 10^4^ cells/mL, whereas the control group shows cell numbers of 5.5 × 10^4^ cells/mL [172]. The hepatocytes cultured in the LCC biocarriers show significantly higher values of albumin secretion and blood urea nitrogen released from the hepatocytes, which indicates a better biocompatibility and higher metabolic activity of cells cultured in LCC biocarriers [91]. The results show that LCC biocarriers are highly biocompatible and can be applied as a precursor of biomaterial for culturing human liver cells [91, 172].

### Other applications

Due to the abundance of hydroxyl groups, LCC can also be applied in composite production as a component in polyurethane polymers and epoxy resins [95]. LCC could be used in polymer composites, as carbohydrate moieties are able to adhere to other ingredients in the polymer system [95].

The combination of rigid, hydrophobic lignin units, and flexible hydrophilic sugar moieties results in a good biological compatibility and strength in LCC material. Softwood and hardwood LCC preparations were successfully tested as biocarrier for liver cells’ culturing. In addition, the high concentration of hydroxyl groups provides an opportunity to employ LCC materials for composite polymer production. However, to our best knowledge, the industrial application of LCCs is still at the research and development stage.

## Conclusions

The compositions, structures, and properties of LCC presented and extracted from different biomass sources are described in this review paper. In softwoods, all lignin moieties are involved in LCC, whereas, in hardwoods and herbaceous plants, LCC constitutes 47–66% and 16–35% of total lignin, respectively. The predominance of benzyl ether linkages is reported in softwood LCC, whereas esters and phenyl glycosidic bonds were found to be dominant in deciduous species. Likewise, in the case of non-wood plants, phenyl glycosidic linkages are dominant. A high amount of benzyl ether and phenyl glycosidic bonds negatively affect kraft pulping and the delignification performance due to alkali resistance of these linkages, while the ester bonds are alkali-liable. Softwood species contain LCC with two different structures of lignin, namely lignin–xylan and lignin–glucomannan. However, hardwood species contain xylan–lignin and cellulose–lignin complexes. Herbaceous LCC mainly contains arabinoxylan linked with lignin moieties via ferulate esters. The application of DMSO/TBAH mixture with Ba(OH)_2_, followed by an enzymatic hydrolysis, allows for the separation of three LCC fractions and is considered to be a universal method of LCC fractionation from biomass. Alkali and combined acid/alkali degradation strategies can be employed for ester and ether linkage analysis. The Smith degradation method is applied for the estimation of phenyl glycosidic bonds of LCC. The application of DDQ and methylation allows for the identification of the bonding sites of sugars involved in benzyl ether and ester linkages. GPC analysis reveals LCC existence in black liquor, hydrolysates, PHL, and SL of NSSC process. ^1^H-NMR technology is applied to research alkali-liable and acid-liable bonds existence in LCC. ^13^C-NMR spectroscopy analysis elucidates the bonding sites of sugar units to lignin moieties. However, for accurate determination and quantification of lignin–carbohydrate linkages, a combined application of 2D HSQC and ^13^C-NMR technologies is required. LCC materials show promising results as anti-HIV agents due to their ability to inhibit HIV adsorption and to penetrate into cells. Moreover, LCCs seem to be efficient as precursors for biocarrier production.

## List of symbols

### Abbreviations and symbols

A. donax: Arundo donax; Ar: aromatic unit; Ara: arabinose; ASGPR: asiaglycoprotein receptors; BE: benzyl ether; C: carbon; C_9_: phenylpropane unit; Carbohydrates-DDQ: carbohydrates liberated from LCC due to DDQ application; CC_50_: cytotoxic concentration; *C*_p_: heat capacity; DDQ: 2,3-dichloro-5,6-dicyanobenzoquinone; DMSO: dimethyl sulfoxide; DMSO-d6: deuterated DMSO; EC_50_: effective concentration; FTIR: Fourier-transform infrared spectroscopy; G_2_: guaiacyl lignin units; Gal: arabinogalactan (Galactan); GC/MS: gas chromatography–mass spectrometry; GGM–L: galactoglucomannan–lignin; GGM–L–Pectin: galactoglucomannan–lignin–pectin; GLC: gas–liquid chromatogram; Glu: glucan; Glu–L: glucan–lignin; Glu–Xyl–L: glucan–xylan–lignin; GM: glucomannan; GM–L: glucomannan–lignin; GM–L–Xyl: glucomannan–lignin–xylan; GPC: Gel permeation chromatography; H: hydrogen; H_2,6_: hydroxyphenyl lignin units; HIV: Human immunodeficiency virus; IR: infrared spectroscopy; IV-DP: intrinsic-differential pressure; L: lignin; L–C bonds: Lignin–carbohydrate bonds; LCC: lignin–carbohydrate complex; LCCs: lignin–carbohydrate complexes; LCC-AcOH: LCC extracted with acetic acid (AcOH); LCC-We: LCC extracted in accordance with the method developed by Watanabe et al. [113]; LFP complex: Lignin–ferulate–polysaccharide complex; Man: mannan; MW: molecular weight; MWL: milled wood lignin; N: nitrogen; ND: not detected; NMR: nuclear magnetic resonance; NSSC: neutral sulphite semichemical process; O: oxygen; PhyGlc: phenyl glycosidic; PHL: prehydrolysis liquor; RI: reflective index; S: sulfur; S_2,6_: syringyl lignin units; SI: selective index; SL: spent liquor; TBAH: tetrabutylammonium hydroxide; T_g_: glass transition temperature; THF-d8: deuterated tetrahydrofuran; *T*_onset_: degradation onset temperature; UV: ultraviolet; Xyl: arabino-4-*O*-methylglucoronoxylan (Xylan); Xyl–Glu–L: xylan–glucose–lignin; Xyl–L: xylan–lignin; Xyl–L–Ara: xylan–lignin–arabinose; Xyl–L–Gal: xylan–lignin–galactan; Xyl–L–Glu: xylan–lignin–glucan; Xyl–L–GM: xylan–lignin–glucomannan; δ_c_: ^13^C NMR chemical shift; δ_H_: ^1^H NMR chemical shift; ^13^C NMR: carbon NMR spectroscopy; ^1^H NMR: proton NMR spectroscopy; 2D HMBC NMR: two-dimensional heteronuclear multiple bond coherence NMR; 2D HSQC NMR: two-dimensional heteronuclear single quantum coherence NMR; 2D TOCSY NMR: two-dimensional total correlation NMR; 3D HSQC-TOCSY NMR: three-dimensional HSQC-TOCSY NMR

### Chemical compounds

(CH_3_CO)_2_O: acetic anhydride; AcOH: acetic acid; Ba(OH)_2_: barium hydroxide; C_2_H_6_OH: ethanol; C_4_H_10_O: ethyl ether; C_5_H_5_N: pyridine; C_6_H_8_O_6_: ascorbic acid; CF_3_SO_3_CH_3_: trifluoromethanesulphonate; CH_3_I: methyl iodide; D_2_O: deuterium oxide; H_2_O: water; H_2_SO_4_: sulphuric acid; H_3_BO_3_: boric acid; HCl: hydrochloric acid; N_2_: nitrogen gas; Na_2_SO_4_: sodium sulfate; NaBH_4_: sodium borohydride; NaH: sodium hydride; NaI: sodium iodide; NaIO_4_: sodium periodate; NaOH: sodium hydroxide
